# Supplementary material for: The KU-PARP14 axis differentially regulates DNA resection at stalled replication forks by MRE11 and EXO1
Source: Nat Commun. 2022 Aug 27;13:5063. doi: 10.1038/s41467-022-32756-5 (PMC9420157; doi:10.1038/s41467-022-32756-5)
Supplement: Supplementary file 1 — Supplementary Information [file 41467_2022_32756_MOESM1_ESM.pdf]

Supplementary Information

The KU-PARP14 axis differentially regulates DNA resection at stalled replication forks by MRE11 and EXO1

Dhoonmoon et al.

Methods (for Supplementary Figures)

Co-immunoprecipitation. Co-immunoprecipitation experiments to investigate the PARP14-MRE11 interaction were performed in HeLa-^{CRISPRa}PARP14 cells, which have increased PARP14 expression through CRISPR activation (CRISPa)-mediated transcriptional induction. To obtain the HeLa-^{CRISPRa}PARP14 cell line, HeLa cells were first transduced with the dCas9 lentiviral construct (Addgene 61425) and selected with 3μg/ml blasticidin. The resulting HeLa-dCas9 cells were then transduced with the lentiviral construct for the MS2-P65-HSF1 (MPH) activator complex (Addgene 61426) and selected with 0.5mg/ml hygromycin. Finally, HeLa-dCas9-MPH cells were transduced with a lentivirus construct containing the guide sequence: TTGCCTTCAGCCTAACACAG (Sigma-Aldrich Custom CRISPR in lentiviral backbone LV06).

For co-immunoprecipitation, HeLa-^{CRISPRa}PARP14 cells were lysed in HEPES lysis buffer (50mM HEPES, 150mM NaCl, 1mM EDTA, 1% TritonX-100, 10% glycerol, 10μM $MgCl_2$) supplemented with cOmplete Protease Inhibitor Cocktail (Roche 11836170001) for 30min at 4°C. Extracts were cleared by centrifugation and incubated with 2μg anti-PARP14 (Santa Cruz Biotechnology sc-377150) or control mouse IgG (GenScript A01007) antibodies overnight at 4°C, followed by incubation with Protein A/G PLUS-agarose beads (Santa Cruz Biotechnology sc-2003) for 2h at 4°C. Beads were washed 5 times with HEPES lysis buffer, and eluted by boiling in Laemmli buffer.

PARP14 in vitro enzymatic assays. To investigate PARP14 catalytic activity *in vitro*, the PARP14 truncation 1470-1801(end) spanning the catalytic PARP domain, was expressed in *E. coli* with an N-terminal 6xHis-tag and purified by NiNTA affinity chromatography. This particular truncation was chosen because a commercially-available GST-tagged PARP14 truncation (purified from insect cells) spanning the same aminoacids was shown to be active in *in vitro*

ADP-ribosylation assays (BPS Bioscience 80514). The 1470-1801(end) fragment, either wildtype or harboring the H1682Q mutation, cloned into the pET plasmid was obtained from VectorBuilder. The plasmids were transformed into *E. coli* BL21(DE3) bacteria cells, and their expression was induced by treatment with 1mM IPTG for 4h at room temperature. Cells were pelleted and lysed in NiNTA lysis buffer (50mM NaH_2PO_4, 300mM NaCl, 10mM imidazole, pH 8.0) for 30mins at 4°C, followed by sonication. Extracts were cleared by centrifugation and incubated with NiNTA agarose beads (ThermoFisher R90115) for 2h at 4°C. Beads were washed with NiNTA wash buffer (50mM NaH_2PO_4, 300mM NaCl, 20mM imidazole, pH 8.0) and eluted in NiNTA lysis buffer (50mM NaH_2PO_4, 300mM NaCl, 250mM imidazole, pH 8.0). Eluted fractions were dialyzed against PBS twice for 24h each.

For *in vitro* ADP-ribosylation reactions, 1μg PARP14 (either commercial GST-PARP14$^{1470\text{-}1801\ \mathbf{WT}}$ as control, or His-PARP14$^{1470\text{-}1801\ \mathbf{WT}}$ and His-PARP14$^{1470\text{-}1801\ \mathbf{H1682Q}}$ purified as described above) was incubated with 25μM biotin-NAD+ (BPS Bioscience 80610) in PARP assay buffer (BPS Bioscience 80602) for 2h at room temperature. The reaction was stopped by boiling in Laemmli buffer and analyzed by western blot with Streptavidin-HRP antibodies (ThermoFisher 21130).

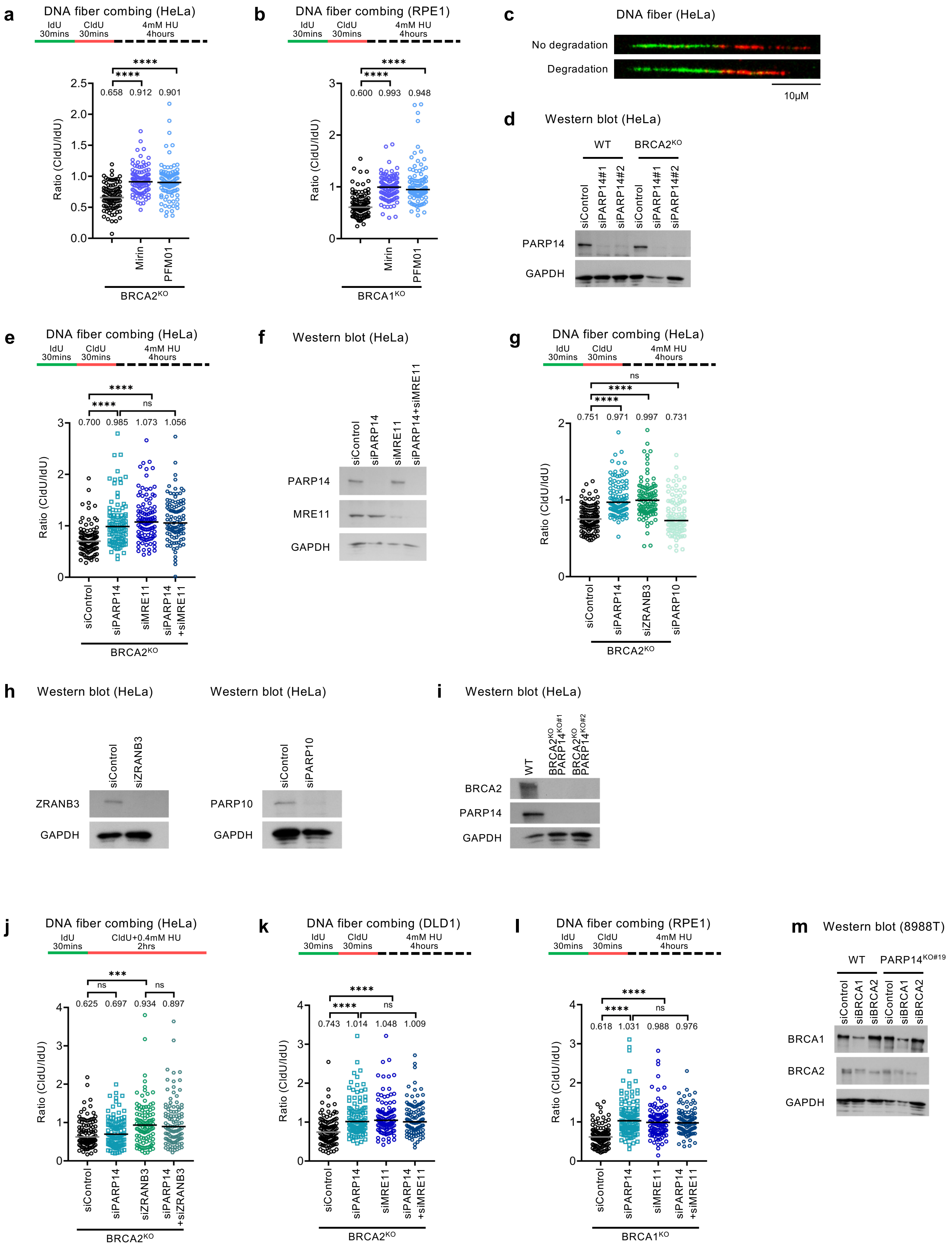
a
DNA fiber combing (HeLa)
IdU 30mins
CldU 30mins
4mM HU 4hours
Ratio (CldU/IdU)
0.658 0.912 0.901
Mirin
PFM01
BRCA2KO
b
DNA fiber combing (RPE1)
0.600 0.993 0.948
BRCA1KO
c
DNA fiber (HeLa)
No degradation
Degradation
10μM
d
Western blot (HeLa)
WT
BRCA2KO
siControl
siPARP14#1
siPARP14#2
PARP14
GAPDH
e
DNA fiber combing (HeLa)
0.700 0.985 1.073 1.056
siControl
siPARP14
siMRE11
siPARP14 +siMRE11
f
Western blot (HeLa)
siPARP14+siMRE11
MRE11
g
DNA fiber combing (HeLa)
0.751 0.971 0.997 0.731
siZRANB3
siPARP10
h
Western blot (HeLa)
ZRANB3
PARP10
i
Western blot (HeLa)
WT
BRCA2KO PARP14KO#1
BRCA2KO PARP14KO#2
BRCA2
j
DNA fiber combing (HeLa)
IdU 30mins
CldU+0.4mM HU 2hrs
0.625 0.697 0.934 0.897
siPARP14 +siZRANB3
k
DNA fiber combing (DLD1)
0.743 1.014 1.048 1.009
l
DNA fiber combing (RPE1)
0.618 1.031 0.988 0.976
m
Western blot (8988T)
PARP14KO#19
siBRCA1
siBRCA2
BRCA1

Supplementary Figure S1. Impact of PARP14 on fork stability in BRCA-deficient cells.

a,b. DNA fiber combing assay showing that inhibition of MRE11 endonuclease activity using PFM01, or inhibition of MRE11 exonuclease activity inhibition using mirin, equally suppresses HU-induced nascent strand degradation in HeLa-BRCA2^{KO} (**a**) and RPE1-BRCA1^{KO} (**b**) cells.

c. Representative micrographs of DNA fiber combing experiments.

d. Western blots showing PARP14 depletion in HeLa cells.

e. DNA fiber combing assays showing that PARP14 and MRE11 knockdowns suppress HU-induced nascent strand degradation in HeLa-BRCA2^{KO} cells.

f. Western blots showing PARP14 and MRE11 depletion in HeLa-BRCA2^{KO} cells.

g. DNA fiber combing assay showing that PARP14 or ZRANB3 knockdown, but not PARP10 knockdown, suppresses HU-induced nascent strand degradation in HeLa-BRCA2^{KO} cells.

h. Western blots confirming knockdowns of ZRANB3 and PARP10 in HeLa-BRCA2^{KO} cells.

i. Western blots confirming the loss of BRCA2 and PARP14 expression in the HeLa-BRCA2^{KO}PARP14^{KO} double knockout cells.

j. DNA fiber combing assay showing that PARP14 knockdown does not affect HU-induced fork slowing in HeLa cells. Knockdown of the DNA translocase ZRANB3, which is essential for fork reversal, is used as a control for defective fork slowing.

k,l. DNA fiber combing assays showing that PARP14 and MRE11 knockdowns suppress HU-induced nascent strand degradation in DLD1-BRCA2^{KO} (**k**) and RPE1-BRCA1^{KO} (**l**) cells.

m. Western blots showing BRCA1 and BRCA2 depletion in 8988T cells.

For all DNA fiber combing assays, the ratio of CldU to IdU tract lengths is presented, with the median values marked on the graphs and listed at the top. At least 100 tracts were quantified for each sample. Asterisks indicate statistical significance (Mann-Whitney, two-tailed).

Source data are provided as a Source Data file.

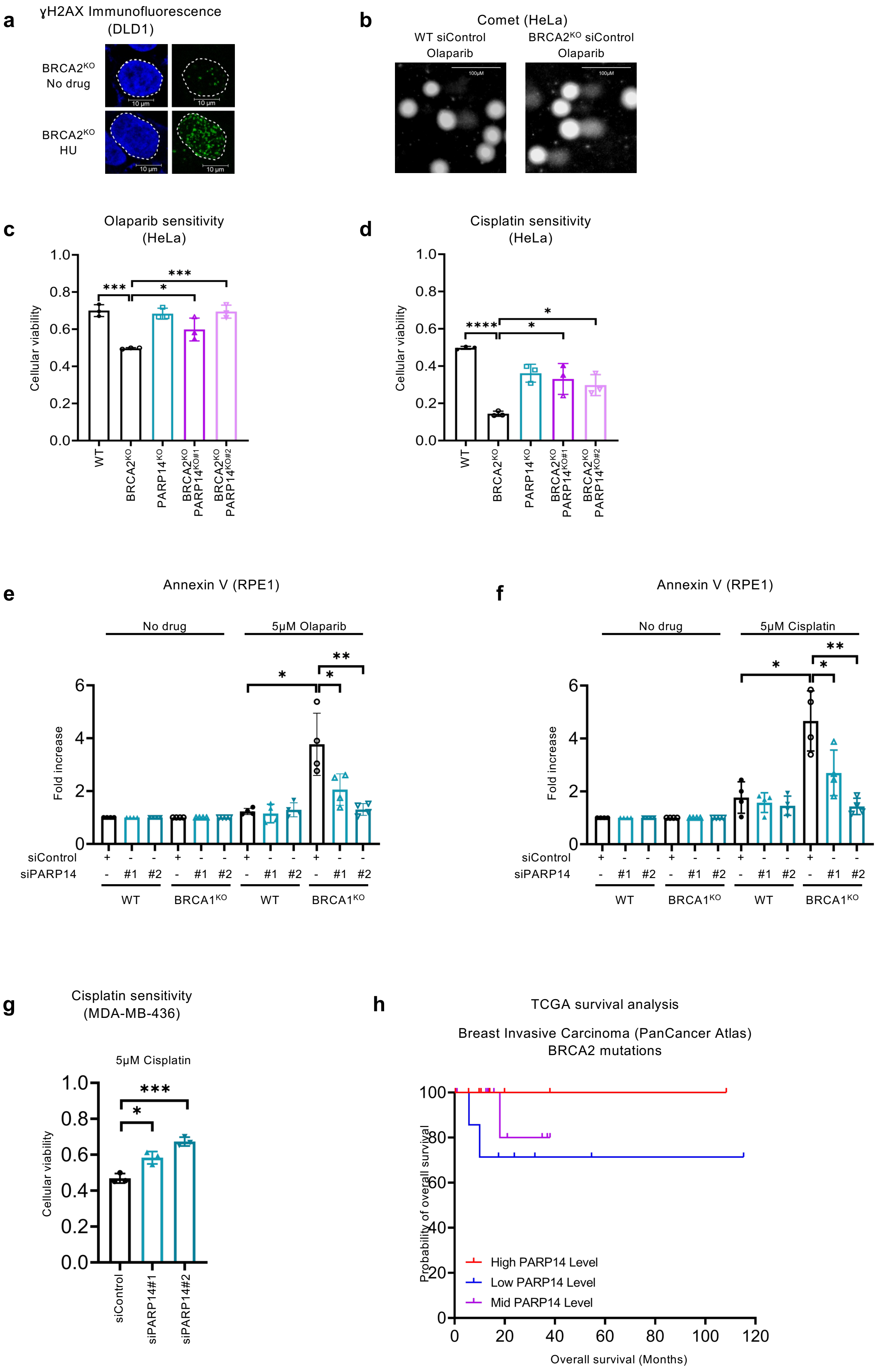
a
ɣH2AX Immunofluorescence (DLD1)
BRCA2KO No drug
BRCA2KO HU
10 μm
b
Comet (HeLa)
WT siControl Olaparib
BRCA2KO siControl Olaparib
100μM
c
Olaparib sensitivity (HeLa)
Cellular viability
1.0
0.8
0.6
0.4
0.2
0.0
***
*
***
WT
BRCA2KO
PARP14KO
BRCA2KO PARP14KO#1
BRCA2KO PARP14KO#2
d
Cisplatin sensitivity (HeLa)
Cellular viability
****
*
*
WT
BRCA2KO
PARP14KO
BRCA2KO PARP14KO#1
BRCA2KO PARP14KO#2
e
Annexin V (RPE1)
No drug
5μM Olaparib
Fold increase
6
4
2
0
*
*
**
siControl
siPARP14
+ - - + - - + - - + - -
- #1 #2 - #1 #2 - #1 #2 - #1 #2
WT
BRCA1KO
WT
BRCA1KO
f
Annexin V (RPE1)
No drug
5μM Cisplatin
Fold increase
*
*
**
siControl
siPARP14
WT
BRCA1KO
WT
BRCA1KO
g
Cisplatin sensitivity (MDA-MB-436)
5μM Cisplatin
Cellular viability
*
***
siControl
siPARP14#1
siPARP14#2
h
TCGA survival analysis
Breast Invasive Carcinoma (PanCancer Atlas)
BRCA2 mutations
Probability of overall survival
100
80
60
40
20
0
High PARP14 Level
Low PARP14 Level
Mid PARP14 Level
0 20 40 60 80 100 120
Overall survival (Months)

Supplementary Figure S2. Impact of PARP14 loss on chemoresistance of BRCA-mutant cells.

a,b. Representative micrographs of γH2AX immunofluorescence (**a**) and neutral comet (**b**) assays.

c,d. Cellular viability assays showing that two independent HeLa-BRCA2^{KO}PARP14^{KO} double knockout cell lines have increased resistance to olaparib (**c**) and cisplatin (**d**) compared to HeLa-BRCA2^{KO} cells. The average of three independent experiments, with standard deviations indicated as error bars, is shown. Asterisks indicate statistical significance calculated using 2-way ANOVA.

e,f. Annexin V assays showing a reduction in olaparib (**e**) and cisplatin (**f**) induced apoptosis upon PARP14 depletion in RPE-BRCA1^{KO} cells. The average of four experiments is presented, with standard deviations shown as error bars. Asterisks indicate statistical significance (t-test two-tailed, unpaired).

g. Cellular viability assay showing that that depletion of PARP14 increases the resistance of MDA-MB-436 cells to cisplatin. The average of three independent experiments (compared to untreated cells), with standard deviations indicated as error bars, is shown. Asterisks indicate statistical significance (t-test two-tailed, unpaired).

h. Analyses of BRCA2-mutant breast invasive carcinoma TCGA cancer datasets showing that high PARP14 levels are associated with increased survival, while low PARP14 levels are associated with reduced survival. Genomic, transcriptomic, and survival data for Breast Invasive Carcinoma samples, part of The Cancer Genome Atlas (TCGA), were obtained from cBioPortal.org. Survival datasets were sorted by BRCA2-status and all BRCA2-mutant samples were used for subsequent analyses. Samples were divided into three groups based on PARP14 expression status in the patient tumor samples: high (0-33rd percentile), middle (33rd-67th percentile) and low (67th-100th percentile). Mantel-Cox log ranked t test was used for statistical

analyses (n=28, p-value= 0.342). The differences observed are not significant, likely because of the small number of BRCA2-mutant samples in the datasets.

Source data are provided as a Source Data file.

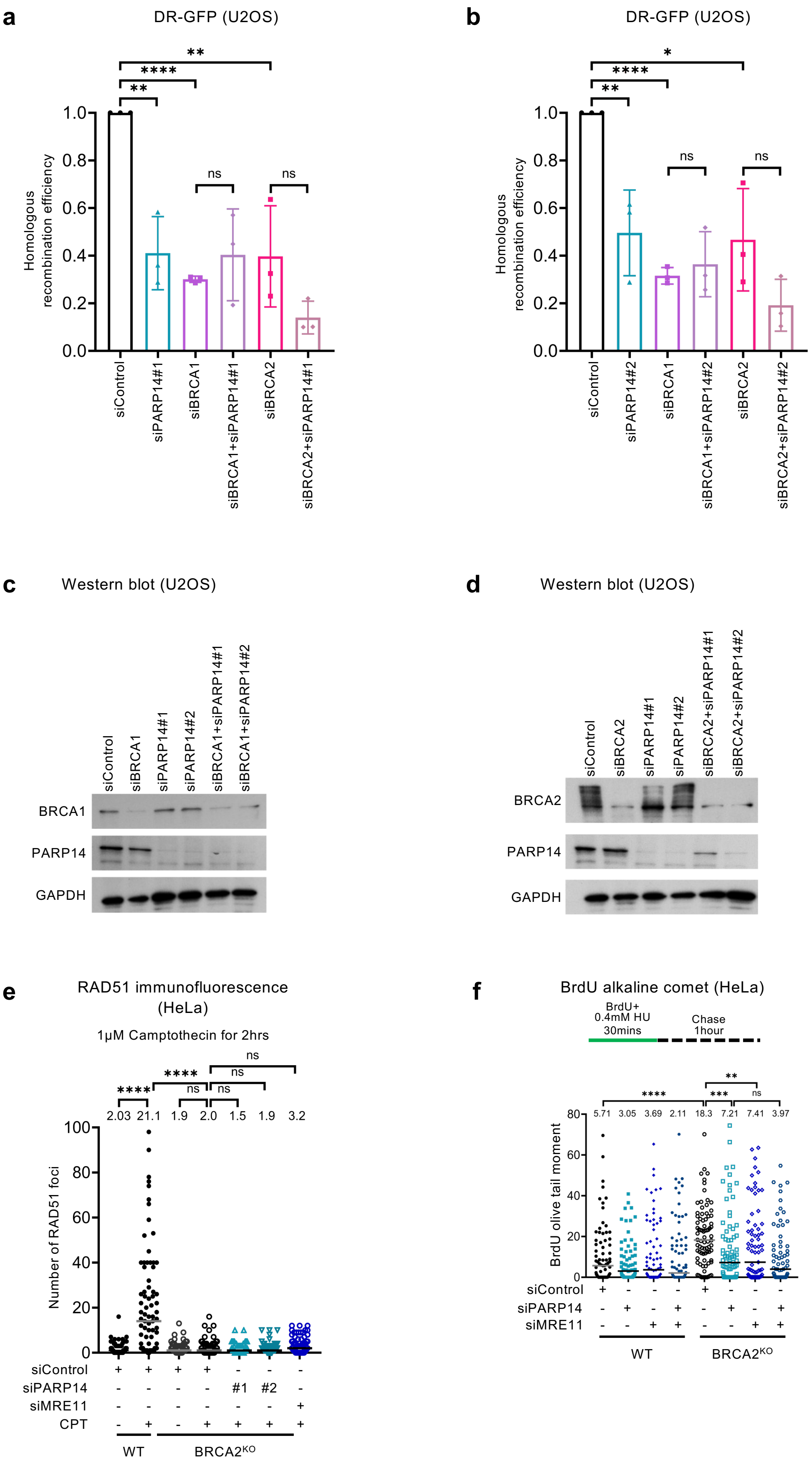
a
DR-GFP (U2OS)
**
****
**
ns
ns
Homologous recombination efficiency
1.0
0.8
0.6
0.4
0.2
0.0
siControl
siPARP14#1
siBRCA1
siBRCA1+siPARP14#1
siBRCA2
siBRCA2+siPARP14#1
b
DR-GFP (U2OS)
*
****
**
ns
ns
Homologous recombination efficiency
1.0
0.8
0.6
0.4
0.2
0.0
siControl
siPARP14#2
siBRCA1
siBRCA1+siPARP14#2
siBRCA2
siBRCA2+siPARP14#2
c
Western blot (U2OS)
siControl
siBRCA1
siPARP14#1
siPARP14#2
siBRCA1+siPARP14#1
siBRCA1+siPARP14#2
BRCA1
PARP14
GAPDH
d
Western blot (U2OS)
siControl
siBRCA2
siPARP14#1
siPARP14#2
siBRCA2+siPARP14#1
siBRCA2+siPARP14#2
BRCA2
PARP14
GAPDH
e
RAD51 immunofluorescence (HeLa)
1μM Camptothecin for 2hrs
ns
****
ns
****
ns
ns
2.03 21.1 1.9 2.0 1.5 1.9 3.2
Number of RAD51 foci
100
80
60
40
20
0
siControl + + + + - - -
siPARP14 - - - - #1 #2 -
siMRE11 - - - - - - +
CPT - + - + + + +
WT
BRCA2KO
f
BrdU alkaline comet (HeLa)
BrdU+ 0.4mM HU 30mins
Chase 1hour
**
****
***
ns
5.71 3.05 3.69 2.11 18.3 7.21 7.41 3.97
BrdU olive tail moment
80
60
40
20
0
siControl + - - - + - - -
siPARP14 - + - + - + - +
siMRE11 - - + + - - + +
WT
BRCA2KO

Supplementary Figure S3. Impact of PARP14 loss on homologous recombination in BRCA-mutant cells.

a-d. DR-GFP assay showing that PARP14 co-depletion with two different siRNAs (**a,b**) does not impact HR efficiency in BRCA1-depleted and BRCA2-depleted cells. The average of three experiments is presented, with standard deviations shown as error bars. Asterisks indicate statistical significance (t-test two-tailed, unpaired). Western blots showing co-depletion of PARP14 with BRCA1 (**c**) and BRCA2 (**d**) are also presented.

e. RAD51 immunofluorescence experiment showing that PARP14 depletion does not affect RAD51 foci formation upon camptothecin treatment in HeLa-BRCA2^{KO} cells. At least 75 cells were quantified for each condition. The mean value is represented on the graphs, and asterisks indicate statistical significance (t-test two-tailed, unpaired).

f. BrdU alkaline comet assay showing that PARP14 depletion reduces replication-associated ssDNA gaps accumulation upon HU treatment in HeLa-BRCA2^{KO} cells. At least 75 nuclei were quantified for each condition. The median values are marked on the graph and listed at the top. Asterisks indicate statistical significance (Mann-Whitney, two-tailed). A schematic representation of the assay conditions is shown at the top.

Source data are provided as a Source Data file.

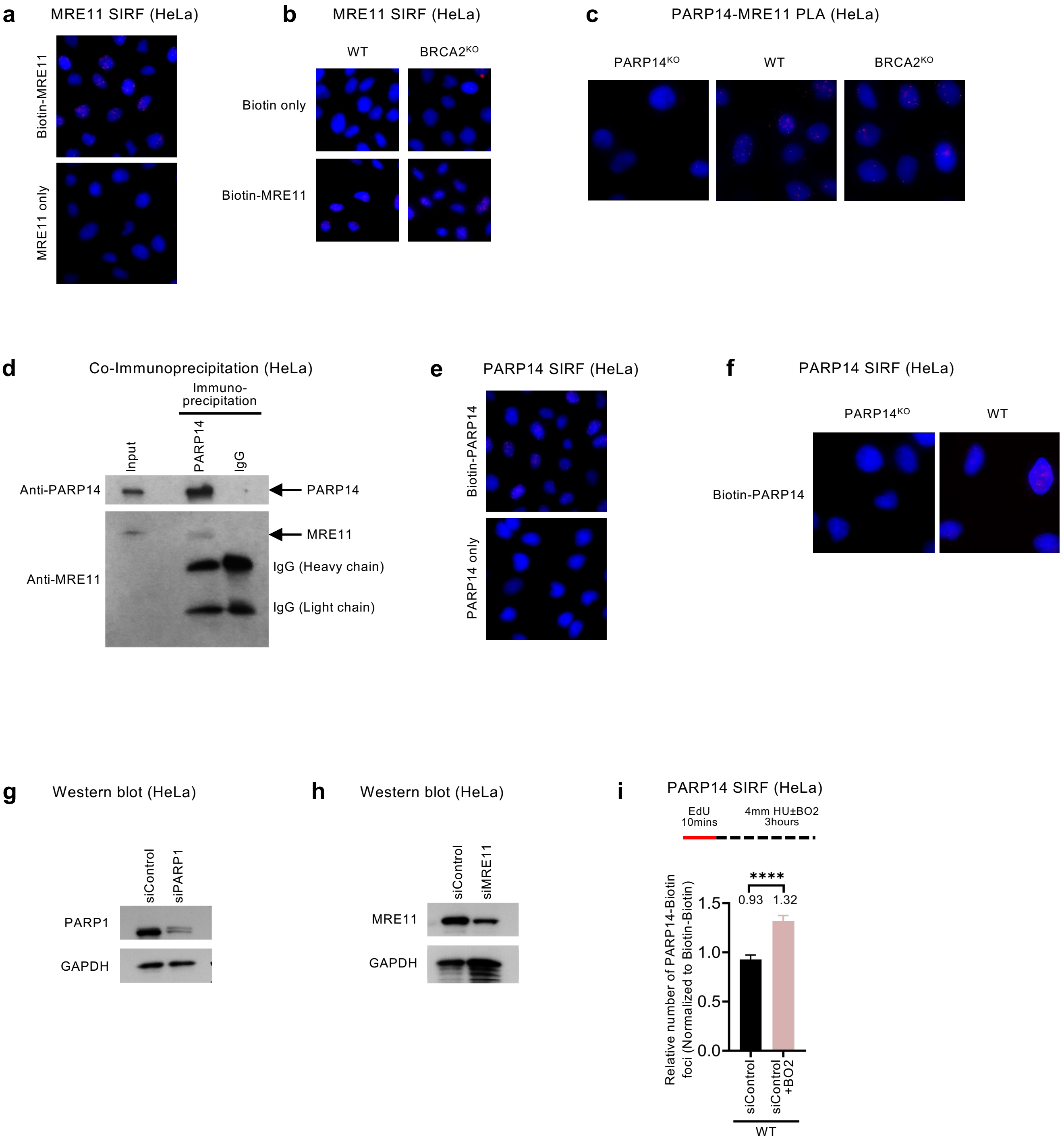
a
MRE11 SIRF (HeLa)
Biotin-MRE11
MRE11 only
b
MRE11 SIRF (HeLa)
WT
BRCA2KO
Biotin only
Biotin-MRE11
c
PARP14-MRE11 PLA (HeLa)
PARP14KO
WT
BRCA2KO
d
Co-Immunoprecipitation (HeLa)
Immuno-
precipitation
Input
PARP14
IgG
Anti-PARP14
PARP14
MRE11
Anti-MRE11
IgG (Heavy chain)
IgG (Light chain)
e
PARP14 SIRF (HeLa)
Biotin-PARP14
PARP14 only
f
PARP14 SIRF (HeLa)
PARP14KO
WT
Biotin-PARP14
g
Western blot (HeLa)
siControl
siPARP1
PARP1
GAPDH
h
Western blot (HeLa)
siControl
siMRE11
MRE11
GAPDH
i
PARP14 SIRF (HeLa)
EdU
10mins
4mm HU±BO2
3hours
Relative number of PARP14-Biotin foci (Normalized to Biotin-Biotin)
****
0.93
1.32
1.5
1.0
0.5
0.0
siControl
siControl +BO2
WT

Supplementary Figure S4. PARP14 binding to MRE11 and nascent DNA.

a. Single antibody SIRF control experiments showing the specificity of the MRE11 SIRF signal in HeLa cells. Representative micrographs showing that the MRE11 SIRF signal is not present when the anti-biotin antibody is not included.

b. Representative micrographs of MRE11 SIRF experiments demonstrating the specificity of the readout, since the MRE11 SIRF signal is not present in HeLa-PARP14^{KO} cells.

c. Representative micrographs of PARP14-MRE11 PLA experiments in HeLa cells. The specificity of the readout is demonstrated by the loss of PARP14-MRE11 PLA foci in HeLa-PARP14^{KO} cells.

d. Co-immunoprecipitation experiments in HeLa-^{CRISPRa}PARP14 cells showing that MRE11 co-precipitates with PARP14.

e. Single antibody SIRF control experiments showing the specificity of the PARP14 SIRF signal in HeLa cells. Representative micrographs showing that the PARP14 SIRF signal is not present when the anti-biotin antibody is not included.

f. Representative micrographs of PARP14 SIRF experiments demonstrating the specificity of the readout, since the PARP14 SIRF signal is not present in HeLa-PARP14^{KO} cells.

g,h. Western blots showing PARP1 (**g**) and MRE11 (**h**) depletion in HeLa cells.

i. PARP14 SIRF experiment showing that treatment with the RAD51 inhibitor B02 induces PARP14 binding to nascent DNA in DLD1 cells. At least 100 cells were quantified for each condition. Bars indicate the mean values, error bars represent standard error of the mean, and asterisks indicate statistical significance (t-test, two-tailed, unpaired). A schematic representation of the assay conditions is shown at the top.

Source data are provided as a Source Data file.

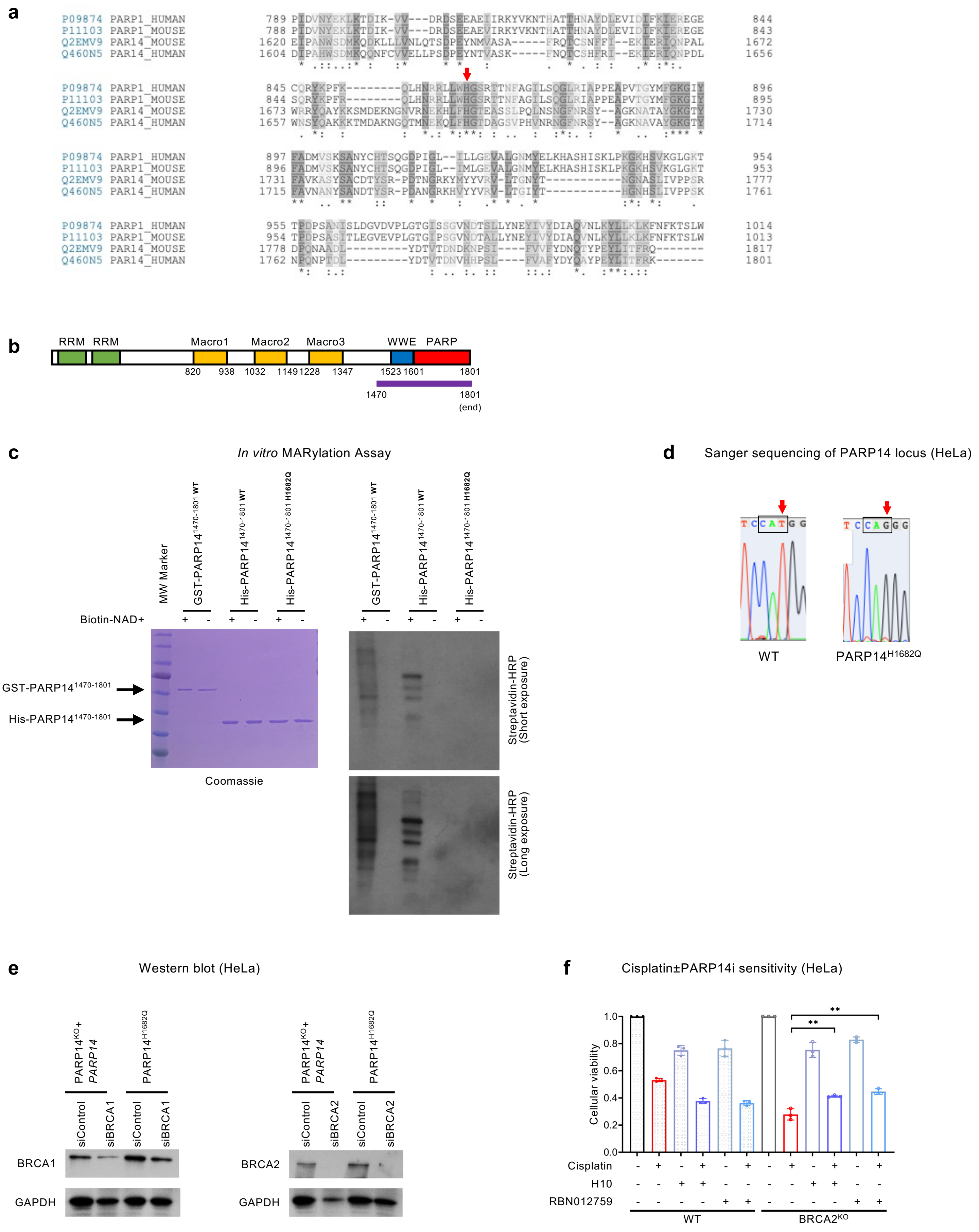

a
P09874 PARP1_HUMAN 789 PIDVNYEKLKTDIK-VV---DRDSEEAEIIRKYVKNTHATTHNAYDLEVIDIFKIEREGE 844
P11103 PARP1_MOUSE 788 PIDVNYEKLKTDIK-VV---DRDSEEAEVIRKYVKNTHATTHNAYDLEVIDIFKIEREGE 843
Q2EMV9 PAR14_MOUSE 1620 EIPANWSDMKQDKLLLVNLQTSDPEYNMVASA-----FRQTCSNFFI--EKIERIQNPAL 1672
Q460N5 PAR14_HUMAN 1604 DIPAHWSDMKQQNFCVVELLPSDPEYNTVASK-----FNQTCSHFRI--EKIERIQNPDL 1656
P09874 PARP1_HUMAN 845 CQRYKPFK--------QLHNRRLLWHGSRTTNFAGILSQGLRIAPPEAPVTGYMFGKGIY 896
P11103 PARP1_MOUSE 844 SQRYKPFR--------QLHNRRLLWHGSRTTNFAGILSQGLRIAPPEAPVTGYMFGKGIY 895
Q2EMV9 PAR14_MOUSE 1673 WRRYQAYKKSMDEKNGNVRNEKHLFHGTEASSLPQLNSNGFNRSY--AGKNATAYGKGTY 1730
Q460N5 PAR14_HUMAN 1657 WNSYQAKKKTMDAKNGQTMNEKQLFHGTDAGSVPHVNRNGFNRSY--AGKNAVAYGKGTY 1714
P09874 PARP1_HUMAN 897 FADMVSKSANYCHTSQGDPIGL--ILLGEVALGNMYELKHASHISKLPKGKHSVKGLGKT 954
P11103 PARP1_MOUSE 896 FADMVSKSANYCHTSQGDPIGL--IMLGEVALGNMYELKHASHISKLPKGKHSVKGLGKT 953
Q2EMV9 PAR14_MOUSE 1731 FAVKASYSACDTYSR-PDTNGRKYMYYVRV-LTGNYT-----------NGNASLIVPPSR 1777
Q460N5 PAR14_HUMAN 1715 FAVNANYSANDTYSR-PDANGRKHVYYVRV-LTGIYT-----------HGNHSLIVPPSK 1761
P09874 PARP1_HUMAN 955 TPDPSANISLDGVDVPLGTGISSGVNDTSLLYNEYIVYDIAQVNLKYLLKLKFNFKTSLW 1014
P11103 PARP1_MOUSE 954 TPDPSASITLEGVEVPLGTGIPSGVNDTALLYNEYIVYDIAQVNLKYLLKLKFNFKTSLW 1013
Q2EMV9 PAR14_MOUSE 1778 DPQNAADL---------YDTVTDNDKNPSI----FVVFYDNQTYPEYLITFRQ------- 1817
Q460N5 PAR14_HUMAN 1762 NPQNPTDL---------YDTVTDNVHHPSL----FVAFYDYQAYPEYLITFRK------- 1801
b
RRM RRM Macro1 Macro2 Macro3 WWE PARP
820 938 1032 1149 1228 1347 1523 1601 1801
1470 1801 (end)
c
In vitro MARylation Assay
MW Marker
GST-PARP14 1470-1801 WT
His-PARP14 1470-1801 WT
His-PARP14 1470-1801 H1682Q
Biotin-NAD+
+ - + - + -
GST-PARP14 1470-1801
His-PARP14 1470-1801
Coomassie
Streptavidin-HRP (Short exposure)
Streptavidin-HRP (Long exposure)
d
Sanger sequencing of PARP14 locus (HeLa)
T C C A T G G
T C C A G G G
WT
PARP14 H1682Q
e
Western blot (HeLa)
PARP14 KO + PARP14
PARP14 H1682Q
siControl siBRCA1 siControl siBRCA1
BRCA1
GAPDH
siControl siBRCA2 siControl siBRCA2
BRCA2
GAPDH
f
Cisplatin±PARP14i sensitivity (HeLa)
Cellular viability
1.0
0.8
0.6
0.4
0.2
0.0
**
**
Cisplatin - + - + - + - + - + - +
H10 - - + + - - - - + + - -
RBN012759 - - - - + + - - - - + +
WT
BRCA2 KO

Supplementary Figure S5. Characterization of the PARP14 H1682Q catalytic inactive mutant.

a. Alignment of the indicated regions of the PARP1 and PARP14 catalytic domains from human and mouse, identifying the conserved His residue in the catalytic domain (H1682 in human PARP14, corresponding to H1698 in mouse Parp14). Alignment was performed using the UniProt Align tool (uniport.org).

b. Diagram showing the domain structure of human PARP14, and indicating the 1470-1801 (end) truncation used for *in vitro* ADP-ribosylation enzymatic assays.

c. *In vitro* ADP-ribosylation enzymatic assays showing that the H1682Q mutation in the PARP14 catalytic domain abolishes the ADP-ribosyltransferase activity. His-tagged PARP14 1470-1801 (end) truncation fragments spanning the catalytic PARP domain, either wildtype (His-PARP14$^{1470\text{-}1801\ \mathbf{WT}}$) or mutant (His-PARP14$^{1470\text{-}1801\ \mathbf{H1682Q}}$) were expressed and purified from bacteria cells as described in the Methods for Supplementary Figures section above. Commercial GST-PARP14$^{1470\text{-}1801\ \mathbf{WT}}$ (expressed and purified from insect cells) was used as control. Both wildtype truncations (commercial GST-tagged, and His-tagged purified by us) showed *in vitro* ADP-ribosyltransferase activity as demonstrated by specific signal (multiple bands) on Streptavidin-HRP blots, corresponding to ADP-ribosylated substrates. This signal is specifically detecting ADP-ribosylation since it does not appear in the absence of biotin-NAD+. These results indicate that, in this *in vitro* assay, the catalytic domain of PARP14 is able to non-specifically ADP-ribosylate contaminating proteins from the purification of the PARP14 truncations. Importantly, the H1682Q mutant showed no Streptavidin-HRP signal, even in the presence of biotin-NAD+, confirming that this mutant is devoid of catalytic activity.

d. Sequencing of the PARP14 genomic locus in the HeLa-PARP14^{H1682Q} mutant cells obtained by CRISPR-mediated genome editing, confirming the H1682Q mutation in all alleles of PARP14. The arrows indicate the T to G mutation (CAT to CAG codon mutation). Trace files were visualized using 4Peaks software (nucleobytes.com).

e. Western blots showing BRCA1 and BRCA2 depletion in HeLa-PARP14^{KO} cells complemented by stable re-expression of PARP14 cDNA, and in in PARP14^{H1682Q} mutant cells.

f. Cellular viability assay showing that that treatment with PARP14 inhibitors H10 (10μM), or RBN012759 (10μM) promotes resistance to cisplatin (10μM) in HeLa-BRCA2^{KO} cells. The average of three independent experiments, with standard deviations indicated as error bars, is shown. Asterisks indicate statistical significance (t-test two-tailed, unpaired).

Source data are provided as a Source Data file.

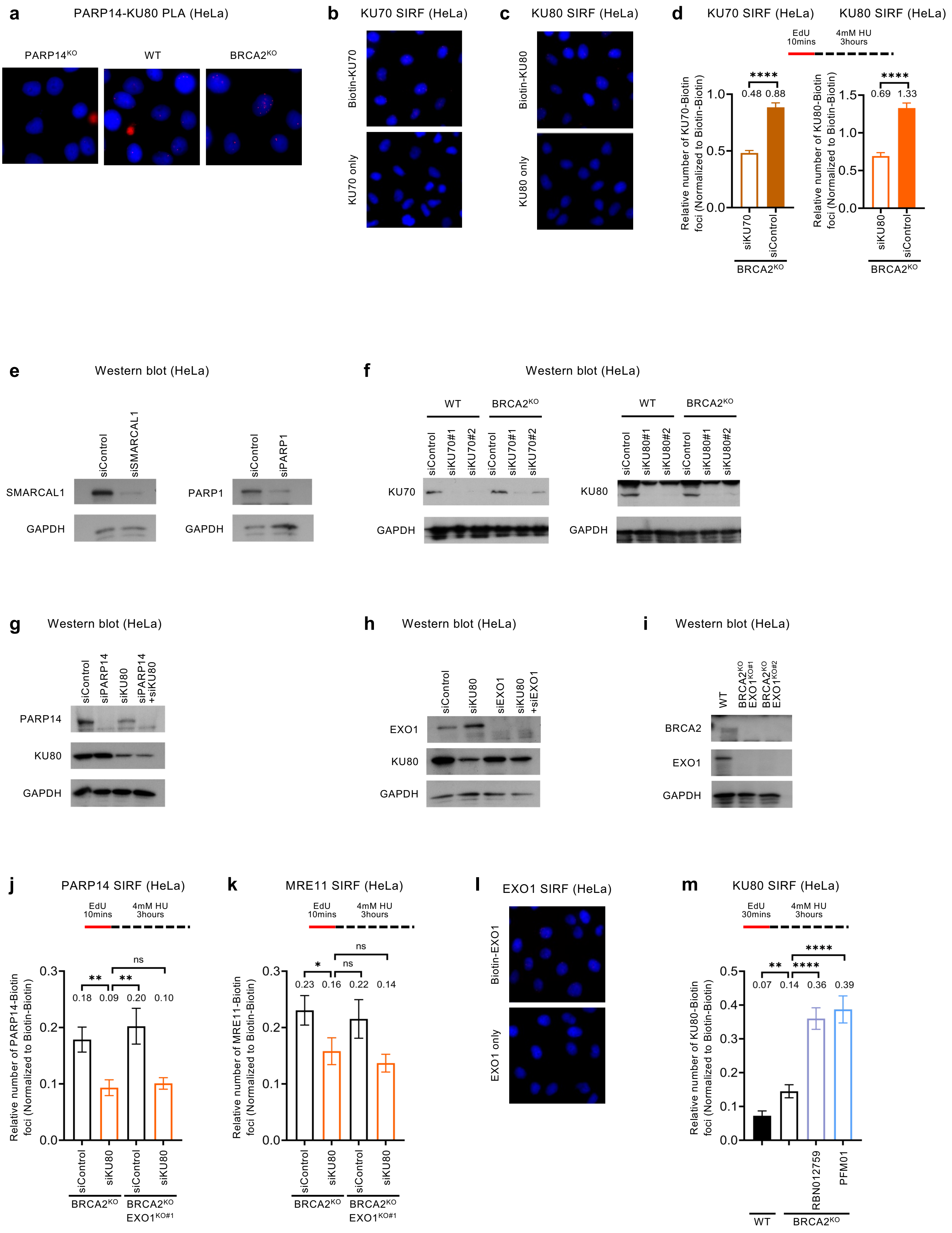

a
PARP14-KU80 PLA (HeLa)
PARP14KO
WT
BRCA2KO
b
KU70 SIRF (HeLa)
Biotin-KU70
KU70 only
c
KU80 SIRF (HeLa)
Biotin-KU80
KU80 only
d
KU70 SIRF (HeLa)
KU80 SIRF (HeLa)
EdU 10mins
4mM HU 3hours
Relative number of KU70-Biotin foci (Normalized to Biotin-Biotin)
Relative number of KU80-Biotin foci (Normalized to Biotin-Biotin)
****
0.48 0.88
0.69 1.33
siKU70
siControl
siKU80
siControl
BRCA2KO
e
Western blot (HeLa)
siControl
siSMARCAL1
siPARP1
SMARCAL1
PARP1
GAPDH
f
Western blot (HeLa)
WT
BRCA2KO
siKU70#1
siKU70#2
siKU80#1
siKU80#2
KU70
KU80
GAPDH
g
Western blot (HeLa)
siPARP14
siKU80
siPARP14 +siKU80
PARP14
h
Western blot (HeLa)
siEXO1
siKU80 +siEXO1
EXO1
i
Western blot (HeLa)
BRCA2KO EXO1KO#1
BRCA2KO EXO1KO#2
BRCA2
j
PARP14 SIRF (HeLa)
Relative number of PARP14-Biotin foci (Normalized to Biotin-Biotin)
**
ns
0.18 0.09 0.20 0.10
k
MRE11 SIRF (HeLa)
Relative number of MRE11-Biotin foci (Normalized to Biotin-Biotin)
*
0.23 0.16 0.22 0.14
l
EXO1 SIRF (HeLa)
Biotin-EXO1
EXO1 only
m
KU80 SIRF (HeLa)
EdU 30mins
0.07 0.14 0.36 0.39
RBN012759
PFM01

Supplementary Figure S6. Impact of PARP14 and MRE11 on nascent DNA binding by KU and EXO1.

a. Representative micrographs of PARP14-KU80 PLA experiments in HeLa cells. The specificity of the readout is demonstrated by the loss of PARP14-KU80 PLA foci in HeLa-PARP14^{KO} cells.

b,c. Single antibody SIRF control experiments showing the specificity of the KU SIRF signal in HeLa cells. Representative micrographs showing that the KU70 (**b**) and KU80 (**c**) SIRF signal is not present when the anti-biotin antibody is not included.

d. KU70 and KU80 SIRF assays validating the specificity of the readout, as the SIRF foci are reduced upon knockdown of the respective KU proteins.

e. Western blots showing SMARCAL1 and PARP1 depletion in HeLa cells.

f. Western blots showing KU70 and KU80 depletion in HeLa cells.

g. Western blots showing co-depletion of PARP14 and KU80 in HeLa cells.

h. Western blots showing co-depletion of EXO1 and KU80 in HeLa cells.

i. Western blots confirming the loss of BRCA2 and EXO1 expression in the HeLa-BRCA2^{KO}EXO1^{KO} double knockout cells.

j,k. PARP14 (**j**) and MRE11 (**k**) SIRF assays showing that KU depletion equally suppresses PARP14 and MRE11 SIRF foci formation in BRCA2^{KO} single knockout cells and BRCA2^{KO}EXO1^{KO} double knockout cells. At least 100 cells were quantified for each condition. Bars indicate the mean values, error bars represent standard error of the mean, and asterisks indicate statistical significance (t-test, two-tailed, unpaired). A schematic representation of the assay conditions is shown at the top.

l. Single antibody SIRF control experiments showing the specificity of the EXO1 SIRF signal in HeLa cells. Representative micrographs showing that the EXO1 SIRF signal is not present when the anti-biotin antibody is not included.

m. KU80 SIRF assays showing that MRE11 or PARP1 inhibition increase KU binding to nascent DNA after 30min EdU labeling in HU-treated HeLa-BRCA2^{KO} cells. At least 100 cells were

quantified for each condition. Bars indicate the mean values, error bars represent standard error of the mean, and asterisks indicate statistical significance (t-test, two-tailed, unpaired). A schematic representation of the assay conditions is shown at the top.

Source data are provided as a Source Data file.
