# Supplementary material for: The KU-PARP14 axis differentially regulates DNA resection at stalled replication forks by MRE11 and EXO1
Source: Nat Commun. 2022 Aug 27;13:5063. doi: 10.1038/s41467-022-32756-5 (PMC9420157; doi:10.1038/s41467-022-32756-5)
Supplement: Supplementary file 3 — Reporting Summary [file 41467_2022_32756_MOESM3_ESM.pdf]

nature portfolio

Corresponding author(s): George-Lucian Moldovan

Last updated by author(s): Aug 1, 2022

Reporting Summary

Nature Portfolio wishes to improve the reproducibility of the work that we publish. This form provides structure for consistency and transparency in reporting. For further information on Nature Portfolio policies, see our Editorial Policies and the Editorial Policy Checklist.

Statistics

For all statistical analyses, confirm that the following items are present in the figure legend, table legend, main text, or Methods section.

n/a	Confirmed	
☐	☒	The exact sample size (*n*) for each experimental group/condition, given as a discrete number and unit of measurement
☐	☒	A statement on whether measurements were taken from distinct samples or whether the same sample was measured repeatedly
☐	☒	The statistical test(s) used AND whether they are one- or two-sided<br>*Only common tests should be described solely by name; describe more complex techniques in the Methods section.*
☒	☐	A description of all covariates tested
☒	☐	A description of any assumptions or corrections, such as tests of normality and adjustment for multiple comparisons
☐	☒	A full description of the statistical parameters including central tendency (e.g. means) or other basic estimates (e.g. regression coefficient) AND variation (e.g. standard deviation) or associated estimates of uncertainty (e.g. confidence intervals)
☐	☒	For null hypothesis testing, the test statistic (e.g. *F*, *t*, *r*) with confidence intervals, effect sizes, degrees of freedom and *P* value noted<br>*Give P values as exact values whenever suitable.*
☒	☐	For Bayesian analysis, information on the choice of priors and Markov chain Monte Carlo settings
☒	☐	For hierarchical and complex designs, identification of the appropriate level for tests and full reporting of outcomes
☒	☐	Estimates of effect sizes (e.g. Cohen's *d*, Pearson's *r*), indicating how they were calculated

Our web collection on statistics for biologists contains articles on many of the points above.

Software and code

Policy information about availability of computer code

Data collection	1) DNA Fiber Combing, Immunofluorescence: LASX 3.5.7.23225<br>2) Proximity Ligation Assay, In-Situ Analysis of Protein Interactions at DNA Replication Forks: SoftWorx 6.5.2<br>3) CometAssay: NIS Elements V1.10.00
Data analysis	1) DNA Fiber Combing: LASX 3.5.7.23225<br>2) Immunofluorescence: ImageJ 1.53a<br>3) Proximity Ligation Assay: ImageJ 1.53a<br>4) In-Situ Analysis of Protein Interactions at DNA Replication Forks: ImageJ 1.53a<br>5) Flow Cytometry: FlowJo V10<br>6) Comet Assay: CometScore 2.0<br>7) Statistics and Data Figure Panel: Graphpad Prism 9 and Microsoft Excel v2205

For manuscripts utilizing custom algorithms or software that are central to the research but not yet described in published literature, software must be made available to editors and reviewers. We strongly encourage code deposition in a community repository (e.g. GitHub). See the Nature Portfolio guidelines for submitting code & software for further information.

Data

Policy information about availability of data

All manuscripts must include a data availability statement. This statement should provide the following information, where applicable:

- Accession codes, unique identifiers, or web links for publicly available datasets
- A description of any restrictions on data availability
- For clinical datasets or third party data, please ensure that the statement adheres to our policy

All data presented in the manuscript is provided as a Source Data file included with the manuscript.

Field-specific reporting

Please select the one below that is the best fit for your research. If you are not sure, read the appropriate sections before making your selection.

☒ Life sciences ☐ Behavioural & social sciences ☐ Ecological, evolutionary & environmental sciences

For a reference copy of the document with all sections, see nature.com/documents/nr-reporting-summary-flat.pdf

Life sciences study design

All studies must disclose on these points even when the disclosure is negative.

Sample size	No statistical method was used to predetermine sample size. Sample size was determined based on previous experiments and relevant literature in the field (eg Taglialatela et al, PMID: 29053959; Lemacon et al, PMID: 29038425; Tirman et al, PMID: 34624216). Statistical significance was obtained with this sample size.
Data exclusions	No data was excluded from the analysis.
Replication	All experiments were performed at least twice. All results were reproducible.
Randomization	Samples were randomly allocated into experimental groups. Samples were processed and analyzed in random order.
Blinding	For imaging-based assays, samples were tested at least once under blinding conditions, and results were reproduced.

Reporting for specific materials, systems and methods

We require information from authors about some types of materials, experimental systems and methods used in many studies. Here, indicate whether each material, system or method listed is relevant to your study. If you are not sure if a list item applies to your research, read the appropriate section before selecting a response.

Materials & experimental systems

n/a	Involved in the study	
☐	☒	Antibodies
☐	☒	Eukaryotic cell lines
☒	☐	Palaeontology and archaeology
☒	☐	Animals and other organisms
☒	☐	Human research participants
☒	☐	Clinical data
☒	☐	Dual use research of concern

Methods

n/a	Involved in the study	
☒	☐	ChIP-seq
☒	☐	Flow cytometry
☒	☐	MRI-based neuroimaging

Antibodies

Antibodies used

Antibodies used for Western blot, at 1:500 dilution, were:
PARP14 (Santa Cruz Biotechnology sc-377150); PARP10 (Novus NB100-2157);
BRCA1 (Santa Cruz Biotechnology sc-6954); BRCA2 (Calbiochem OP95);
ZRANB3 (Invitrogen PA5-65143);
PARP1 (Cell Signaling Technology 9542S); MRE11 (Santa Cruz Biotechnology sc-135992); KU70 (Abcam ab83501);
KU80 (Abcam ab119935);
EXO1 (Novus NBP2-26392); SMARCAL1 (Invitrogen PA5-54181); PARP1 (Cell Signaling Technology 9542).
SMARCAL1 (Invitrogen PA5-54181);
GAPDH (Santa Cruz Biotechnology sc-47724).

Antibodies used for immunofluorescence were: γH2AX antibody (Millipore Sigma JBW301).

Antibodies used for DNA fiber combing were: CldU (Abcam 6236), IdU (BD 347580), and DNA (Millipore Sigma MAD3034) and secondary Cy3 (Abcam 6946), Cy5 (Abcam 6565), or BV480 (BD Biosciences 564879) conjugated antibodies.

Antibodies used for proximity ligation assays were PARP14 (Santa Cruz Biotechnology sc-377150); MRE11 (Genetex GTX70212); KU70 (Santa Cruz Biotechnology sc-56092); KU80 (Abcam ab-119935); Biotin (mouse: Jackson ImmunoResearch 200-002-211; rabbit: Bethyl Laboratories A150-109A), EXO1 (Santa Cruz Biotechnology sc-56092).

Validation	Antibodies were validated by western blots or imaging-based assays using siRNA-mediated knockdown or CRISPR-mediated knockout. The data is presented in the manuscript (Figs. 3i, 4a, 4c, 5a, 7a, 8e, 8f; Supplementary Figs. S1d, S1f, S1h, S1i, S1m, S2a, S3c, S3d, S4a, S4b, S4c, S4e, S4f, S4g, S4h, S5e, S6a, S6b, S6c, S6d, S6e, S6f, S6g, S6h, S6i, S6j, S6k, S6l.

Eukaryotic cell lines

Policy information about cell lines

Cell line source(s)	HeLa and U2OS cells were obtained from ATCC. 8988T-PARP14KO cells, HeLa-PARP14KO cells, and HeLa- BRCA2KO cells were generated in our laboratory and previously described. DLD1 and DLD1-BRCA2KO cells (Horizon HD105-007) were obtained from Dr. Robert Brosh (National Institute on Aging, Baltimore, MD). 8988T, RPE1 and RPE1-BRCA1KO (also harboring p53 homozygous deletion) were obtained from Dr. Alan D'Andrea (Dana-Farber Cancer Institute, Boston, MA). U2OS DR-GFP cells were obtained from Dr. Jeremy Stark (City of Hope National Medical Center, Duarte, CA). MDA- MB-436 cells were obtained from Dr. Hong-Gang Wang (Penn State College of Medicine).
Authentication	Authentication was performed regularly based on morphology and gene/protein expression (in case of genetic alterations)
Mycoplasma contamination	Cell lines tested negative for mycoplasma
Commonly misidentified lines (See ICLAC register)	None of the cell lines used are listed on the ICLAC register version 10
